# Curcumin Increases the Pathogenicity of *Salmonella enterica* Serovar Typhimurium in Murine Model

**DOI:** 10.1371/journal.pone.0011511

**Published:** 2010-07-09

**Authors:** Sandhya A. Marathe, Seemun Ray, Dipshikha Chakravortty

**Affiliations:** Centre for Infectious Disease Research and Biosafety Laboratories, Department of Microbiology and Cell Biology, Indian Institute of Science, Bangalore, India; University of Hyderabad, India

## Abstract

Curcumin has gained immense importance for its vast therapeutic and prophylactic applications. Contrary to this, our study reveals that it regulates the defense pathways of *Salmonella enterica* serovar Typhimurium (*S*. Typhimurium) to enhance its pathogenicity. In a murine model of typhoid fever, we observed higher bacterial load in Peyer's patches, mesenteric lymph node, spleen and liver, when infected with curcumin-treated *Salmonella*. Curcumin increased the resistance of *S*. Typhimurium against antimicrobial agents like antimicrobial peptides, reactive oxygen and nitrogen species. This increased tolerance might be attributed to the up-regulation of genes involved in resistance against antimicrobial peptides - *pmrD* and *pmrHFIJKLM* and genes with antioxidant function - *mntH*, *sodA* and *sitA*. We implicate that iron chelation property of curcumin have a role in regulating *mntH* and *sitA*. Interestingly, we see that the curcumin-mediated modulation of *pmr* genes is through the PhoPQ regulatory system. Curcumin downregulates SPI1 genes, required for entry into epithelial cells and upregulates SPI2 genes required to intracellular survival. Since it is known that the SPI1 and SPI2 system can be regulated by the PhoPQ system, this common regulator could explain curcumin's mode of action. This data urges us to rethink the indiscriminate use of curcumin especially during *Salmonella* outbreaks.

## Introduction

In the last few years, curcumin has been widely used as an herbal medicine [1], [2]. Curcumin's anti-bacterial activity *in vitro* (against *S. aureus, E.coli, S. lutea*) has been attributed to its phototoxic effect which produces H_2_O_2,_ a toxic moiety for the bacteria [3]. Same study reported that *Salmonella enterica* serovar Typhimurium (*S.* Typhimurium) is susceptible but more resistant to curcumin than *S*. *aureus* [3]. In *B. subtilis,* curcumin inhibits FtsZ assembly dynamics by perturbing its GTPase activity [4]. It affects the growth of *H. pylori* by inhibiting shikimate dehydrogenase, a vital enzyme [5]. However, curcumin protects *S.* Typhimurium TA1535/pSK1002 and *E. coli* K-12 strains by inhibiting SOS induction and mutagenesis by UV light [6]. Curcumin also confers resistance to *E. coli*, *B. megaterium*, and *B. pumilus* against the inactivation of DNA, induced by gamma radiation [7]. The knowledge of the effect of curcumin on pathogenicity of bacteria is still in its infancy. In our work, we have addressed the role of curcumin on pathogenicity of *S*. Typhimurium, a Gram-negative facultative intracellular pathogen that causes systemic disease in mice similar to the typhoid fever caused by serovar Typhi in humans. *S*. Typhimurium is an important causative agent of gastroenteritis in humans. The pathogenicity of *S*. Typhimurium is largely dependent on its ability to evade and resist host innate factors like antimicrobial peptides (AMPs), reactive oxygen (ROS) and nitrogen species (RNI) [8], [9], [10], [11], [12] encountered during its pathogenic life cycle. Presence of scavengers [13], antioxidant enzymes [13], iron and manganese transport systems [14], LPS modification systems [15], proteases, DNA repair systems [13] etc. help the bacteria to protect themselves from the innate immune factors of the host. *S*. Typhimurium have acquired *Salmonella* Pathogenicity Island 1 (SPI1) and *Salmonella* Pathogenicity Island 2 (SPI2) that play an important role in the entry and survival of bacteria inside the host cells, respectively [16].

In the following study, we demonstrate that curcumin enhances the pathogenicity of *S*. Typhimurium in murine typhoid model. We have attempted to delineate the pathway for the same. We show that curcumin increases the resistance of *S*. Typhimurium against the antimicrobial defenses viz. AMPs and oxidative stress, exerted by the host. We further implicate that curcumin modulate the expression of antioxidant genes through its iron chelation property and SPI2 and *pmr* (LPS modification) genes through PhoPQ two component system.

## Materials and Methods

### Bacterial strains, media and growth conditions

*S*. Typhimurium strain 12023 was used as wild-type (WT). WT, WT with GFP (pFPV25.1) *phoP^-^*(*phoPQ* mutant, CS015 [17]) (Kind gift from Prof. M. Hensel, Institut fűr Klinische Mikrobiologie, Germany) and *ΔsitA*, generated in this study [18] were grown at 37°C in Luria broth (LB) containing 50 µg/ml nalidixic acid, ampicillin, 20 µg/ml chloramphenicol and 50 µg/ml kanamycin respectively. Curcumin (20 µM; Sigma), ferric chloride (32 µg/ml) and ferrous sulphate (20 µM) were added to the media wherever required. Alternatively, the bacteria were also sub-cultured and grown in F-media [19] for observing growth pattern.

### Construction of *sitA* deletion mutant in *S.* Typhimurium

One step inactivation strategy described by Datsenko and Wanner [18] was used. Briefly, *S*. Typhimurium strain carrying a red helper plasmid (pKD46) were grown in LB with 50 µg/ml ampicillin and 10 mM L-arabinose at 30°C to an OD_600 nm_ of 0.3–0.4. The electrocompetent cells were prepared by harvesting the cells and then washing two times with sterile MilliQ water and then with ice-cold 10% (v/v) glycerol. The PCR product containing the kanamycin-resistance gene (from plasmid pKD4) flanked by sequences upstream and downstream of *sitA* was electroporated into electrocompetent cells. The knockout was selected for kanamycin resistance and confirmed by PCR using the confirmatory primers. The knockout obtained was non-polar.

### Cloning of *sitA* gene in *ΔsitA*

The *sitA* gene was amplified using the cloning primers (Supplementary table S1) and *S*. Typhimurium genomic DNA. The amplified product was digested with BamHI and HindIII. The digested product was purified and ligated with the predigested pQE60 plasmid. The product was transformed into competent *E.coli* cells and positive colonies were selected. Plasmid was isolated from the positive clone and transformed into *ΔsitA* strain to obtain the complement.

### Construction of *lacZ* transcriptional fusions and β-galactosidase assay

*LacZ* transcriptional fusions to *mntH, sitA, sodA,* and *hilA* promoters were constructed as described previously [20] using primers listed in supplementary table S1. *pmrD, pmrHFIJKLM (pmrHM) and spiC* lacZ construct were kindly provided by Vidya Negi and Priyanka Das (our lab). Transcriptional activity of each gene was determined by performing β-galactosidase assay as described previously [21].

### Eukaryotic cell lines and growth conditions

RAW 264.7 cells were a kind gift from Prof. Anjali Karande (Department of Biochemistry, IISc, Bangalore). Intestine 407 and Caco-2 cells were kind gift from Dr. Patole (National Center for Cell Science, Pune). BMDM were isolated from NRAMP1^+/+^ mice (SWISS ALBINO) as described previously [20]. The cells were grown in Dulbecco's Modified Minimum Essential Medium supplemented with 10% fetal bovine serum and maintained at 37°C and 5% CO_2_. For Caco-2 cells, 1% non-essential amino acids was added to the medium.

### Intracellular survival assay

The gentamicin protection assay was performed as described previously [8].

### Immunoblot

Curcumin treated and untreated bacteria were subcultured in F-media (pH 5). Bacteria were pelleted at different time points and lysed in SDS lysis buffer. Equal amount of protein was processed for immunoblotting using anti-SseB antibody (a kind gift from Prof. Michel Hensel).

### H_2_O_2_ and NO^.^ tolerance assay

H_2_O_2_ and NO^.^ tolerance tests were done as described by Buchmeier *et al.* [22] and Chakravortty *et al.* [23] respectively, with some modifications. Briefly, overnight cultures of the wild type *S.* Typhimurium grown under different conditions were diluted to 10^5^ CFU in F-media (pH 5) and incubated with 1 mM H_2_O_2_ or NaNO_2_ at 37°C and 180 rpm for 4 h. NaNO_2_ (NaNO_2_, Sigma-Aldrich) and hydrogen peroxide (30% H_2_O_2_, Qualigens) solutions were prepared freshly. Serial dilutions were made and plated onto LB agar plates for the enumeration of surviving bacteria.

### Antimicrobial peptide sensitivity assay

The assay was done as described by Fields *et al.* [24], with some modifications. Bacteria, in the exponential phase (3 h incubation at 37°C, 180 rpm, after 1∶33 dilution of overnight culture in LB) were taken for the assay. Bacteria (2–5×10^5^ for CFU analysis and 5–7.5×10^6^ for flow cytometry) were diluted in 0.5% tryptone-0.5% sodium chloride solution and incubated with AMPs [polymyxin B, 1 µg/ml (for CFU analysis), 0.5 µg/ml (Flow cytometry) and protamine, 50 µg/ml (for CFU analysis), 30 µg/ml (Flow cytometry)] at 37°C and 180 rpm. After 1 h, the samples were either plated on LB agar for the enumeration of surviving bacteria or were incubated with 1 µg/ml of bis-(1,3-dibutylbarbituric acid)-trimethine oxonol [DiBAC_4_(3); Invitrogen] for 10 min followed by 2 washes with PBS. The DiBAC_4_ (3) treated samples were further analysed in FACS scanner (BD Biosciences) to test for the change in membrane permeability upon AMP treatment.

### Bacterial RNA extraction and RT-PCR analysis

RNA was isolated from the log phase bacteria grown in LB with and without curcumin. cDNA was prepared from the bacterial RNA using a reverse transcription system (Fermentas). cDNA was amplified (35 cycles or 25 cycles for *16S rRNA*) using primers (supplementary Table S1) that amplify the intergenic region of the specific genes.

### Mice experiment

4–6-weeks-old BALB/c mice (obtained from Central Animal Facility, Indian Institute of Science, Bangalore, India) were maintained under specific-pathogen-free conditions. All the procedures with animals were carried out in accordance with approved protocols of Indian Institute of Science, Bangalore. Mice were infected intra-gastrically or intra-peritoneally with 10^7^ or 10^3^ CFU of *S.* Typhimurium respectively. 3 days after infection, liver, spleen, mesenteric lymph nodes (MLN), Peyer's patches (PP) and kidney were isolated under aseptic conditions, weighed and homogenized in a tissue homogenizer. The homogenate was plated at different dilutions to get CFU per gram weight of organ.

For invasion assay, the mice were sacrificed 1 h post-infection. PP were isolated aseptically, weighed, homogenized and plated at different dilutions to determine the CFU.

### Mucus sensitivity test

Mucus was recovered from the small intestine of the BALB/c mice and diluted (2 times) with PBS to reduce the viscosity. The suspension was then centrifuged at 1000 xg for 5 min to settle the cellular debris. 10^7^ bacteria from overnight culture were incubated in mucus under shaking condition (180 rpm) at 37°C. After 1 h, the suspensions were plated onto *Salmonella Shigella*-agar to determine the amount of surviving bacteria per milligram of the mucus protein.

### Statistical analysis

Each experiment was performed in triplicate and repeated a minimum of 3 times. Data are shown as means ± SE. Student's *t*-test or Mann Whitney U test were used for data analysis. All the analyses were done with Sigma plot (version 10) or Graph Pad Prism (version 5) softwares. Wherever applicable, *P* value ≤0.05 was considered as statistically significant.

## Results

### Presence of curcumin during growth of *S.* Typhimurium increases its rate of proliferation

**Figure 1:**
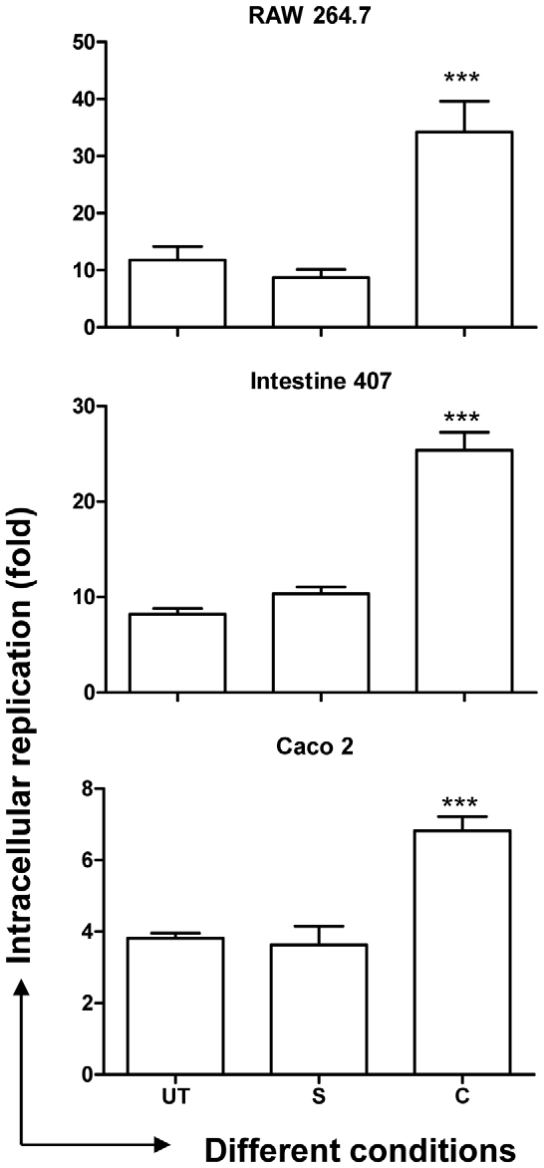
Fold proliferation (2 h to 16 h) of *S.* Typhimurium. The cells (RAW 264.7, Intestine 407 & Caco-2) infected with solvent (S), curcumin (C, 20 µM) treated or untreated (UT) *S.* Typhimurium were lysed at 2 h & 16 h post infection and fold replication of the bacteria was calculated. *** P<0.001.

The concentration of curcumin was fixed at 20 µM based on the MTT assay for eukaryotic cells and growth curve for bacterial cells (Supplementary Figure S1, Materials and Methods S1). We first assessed the effect of curcumin on pathogenicity of *S*. Typhimurium by intracellular survival assay in macrophages and epithelial cells. The fold-proliferation, from 2 h to 16 h post infection, of *S*. Typhimurium grown in the presence of curcumin (curcumin-treated) was 2–3 fold higher compared to that of the untreated (Figure 1) in RAW 264.7, Intestine 407 and Caco-2 cells. These results suggest that curcumin treatment aids survival of *S*. Typhimurium inside the host cells.

**Figure 2:**
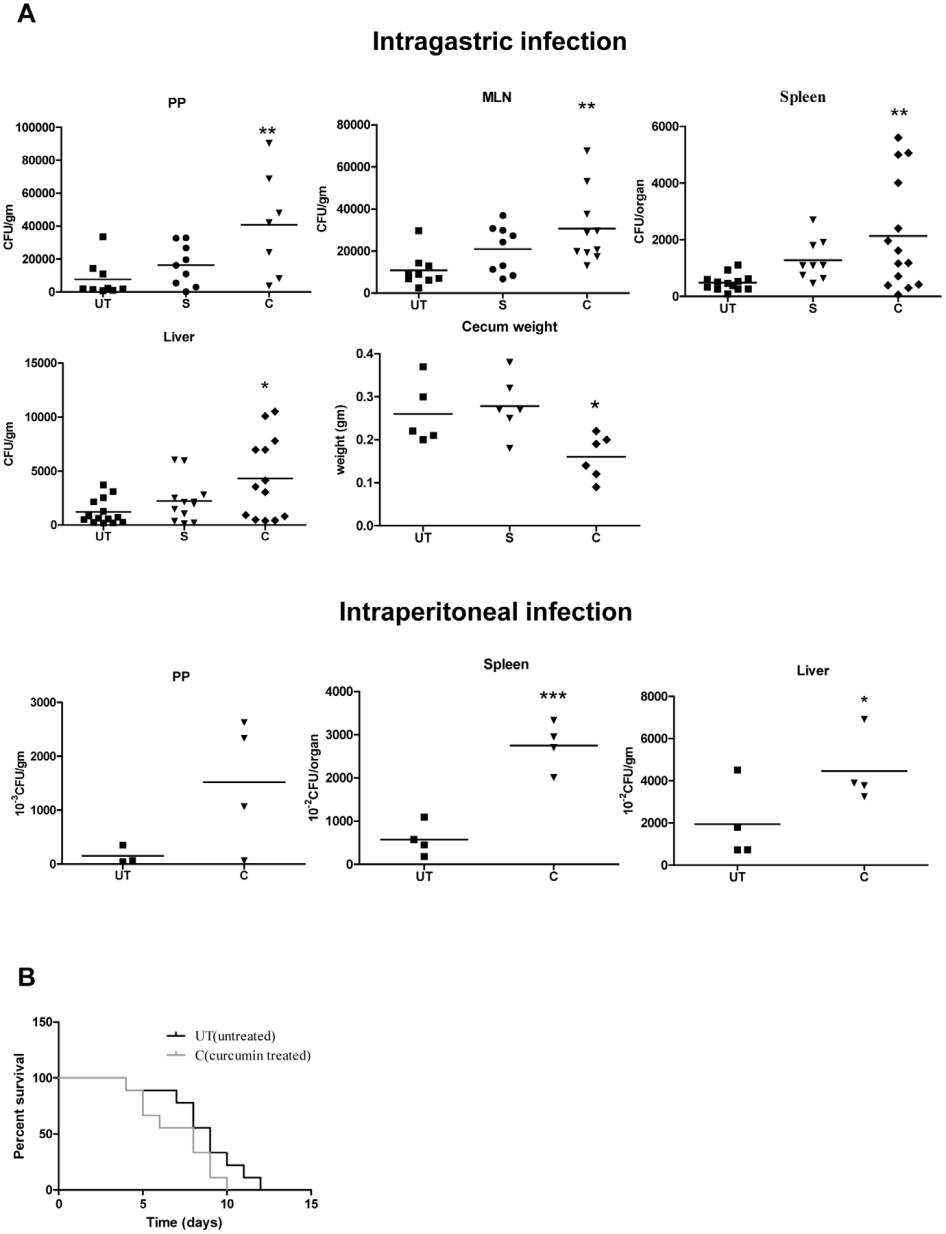
Curcumin treated *S.* Typhimurium showed enhanced virulence in murine model of typhoid fever. **A.** Bacterial load in different organs of the mice infected with solvent (S) curcumin (C, 20 µM) treated or untreated (UT) *S*. Typhimurium. 3-days post-infection, different organs of infected mice were aseptically isolated, weighed, homogenized and plated to get the CFU/gm. Cecum weight was plotted for mice infected intragastrically. **B.** Survival of mice after infection with either curcumin treated or untreated *S*. Typhimurium. ** 0.001≤P<0.01 and * 0.01≤P<0.05.

BALB/c mice infected intragastrically or intraperitoneally with curcumin-treated or untreated *S*. Typhimurium were either monitored for survival or dissected 3 days post infection to analyse the bacterial burden in PP, MLN, spleen and liver. The mice infected with curcumin treated *S*. Typhimurium had significantly more bacterial burden in the organs tested (Figure 2A) and showed lower survival rate (Figure 2B). The cecum weight (a hallmark of salmonellosis) [25] of the mice infected (intragastrically) with curcumin-treated *S*. Typhimurium was significantly less than that of the mice infected with untreated *S*. Typhimurium (Figure 2A). These observations strongly show that curcumin increases the pathogenicity of *S*. Typhimurium.

### Curcumin treatment altered the expression of SPI2 genes of *S.* Typhimurium

Inside the host cell, *S*. Typhimurium exists in a compartment known as *Salmonella* containing vacuole (SCV) [26]. SCV has pH of 5, low Mg^2+^ concentration, low phosphate concentration, high K^+^ concentration, and limited amino acid availability. F-medium mimics to some extent the SCV environment [27]. To test whether curcumin treatment gives any growth advantage to *S*. Typhimurium under the stringent conditions of SCV, the growth pattern of curcumin-treated bacteria was assessed in F-media. There was no significant difference in the growth pattern of curcumin-treated and untreated *S*. Typhimurium in F-media (supplementary Figure S2), implying that curcumin does not increase the proliferation of *S*. Typhimurium in SCV but might be increasing its resistance against the host antimicrobial agents.

**Figure 3:**
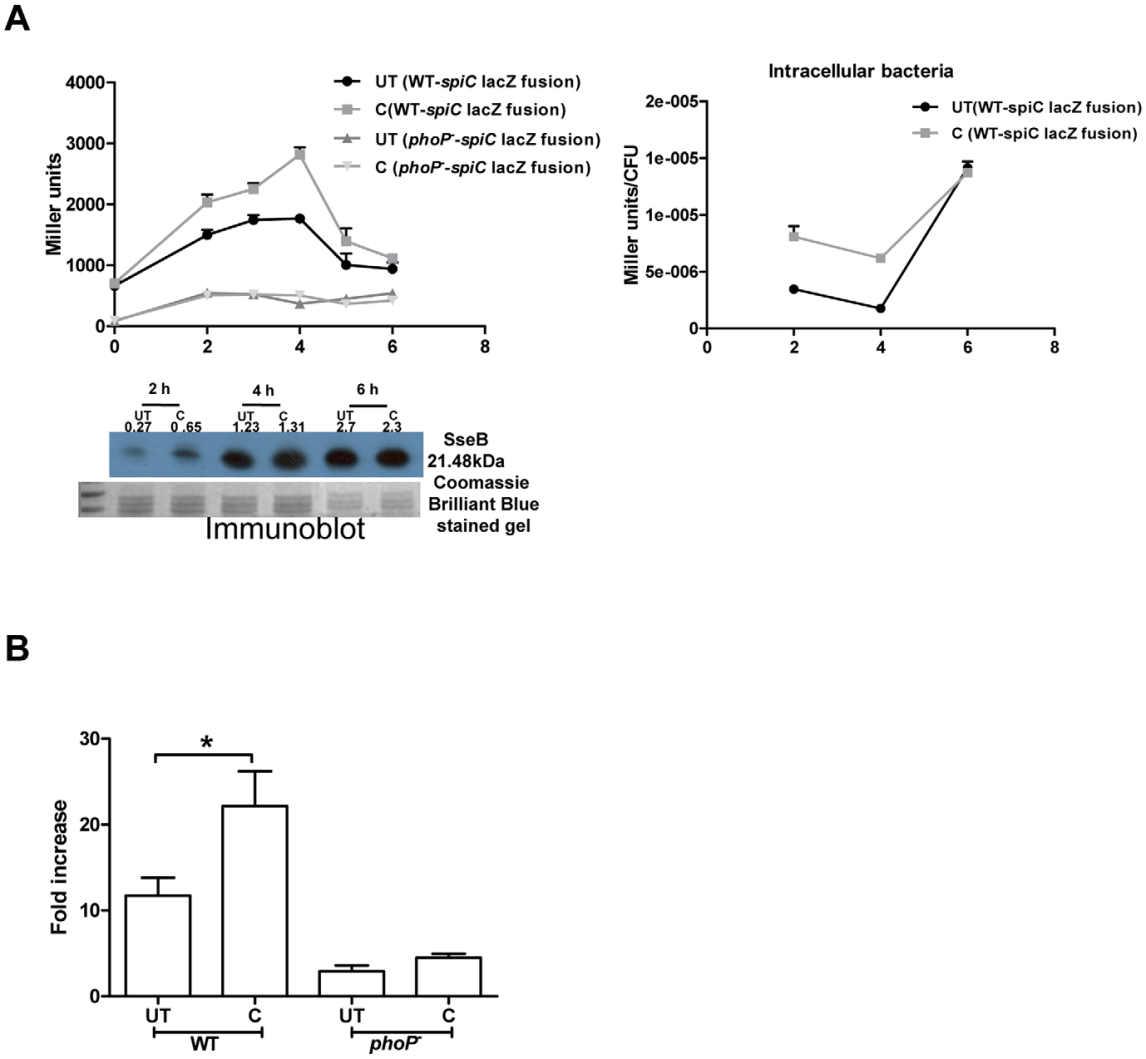
Modulation of SPI2 genes by curcumin. **A.** Promoter assay for *spiC* gene using a reporter strain (WT harbouring lacZ fusion of *spiC* promoter in pHG86 plasmid) and immunoblot for SseB protein. β-galactosidase assay was done at different time points for bacteria, subcultured (1∶33) from overnight culture in F-media (pH 5) or isolated from RAW 264.7 cells. **B.** Fold proliferation of WT and *phoP^-^ S*. Typhimurium. RAW 264.7 infected with curcumin (C, 20 µM) treated or untreated (UT) *S*. Typhimurium were lysed after 2 h & 16 h and fold replication of the bacteria was calculated.

To defend itself from the host antimicrobial agents, *S*. Typhimurium uses SPI2 effector proteins [27], [28]. SPI2 genes play an important role in the intracellular survival of bacteria, especially in macrophages [23], [28] by preventing the fusion of the NADPH oxidase containing vesicles with the SCV [23], [29], [30]. SpiC, is a SPI2 protein that is necessary for the translocation of SPI2 effectors into infected macrophages [31] and preventing the fusion of SCV with endosome/lysosome [32]. Inside the host, curcumin-treated bacteria survive better than the untreated ones. So the regulation of SPI2 genes, if any, by curcumin was assessed by reporter assay and western blotting. The promoter activity of *spiC* was examined in curcumin treated and untreated bacteria, grown in F media or isolated from RAW 264.7 cells. For this *S*. Typhimurium harboring a *spiC-lacZ* construct (*spiC* promoter cloned upstream of promoterless lacZ gene in pHG86 plasmid) was used. The β-galactosidase assay was performed to check for the change in promoter activity, if any. The increased β-galactosidase activity in curcumin treated cells indicates that curcumin increased the promoter activity of *spiC* at early time points (upto 4–5 h) after which the activity remained similar to that of the untreated bacteria (Figure 3A). Immunoblot for SseB, a SPI2 encoded translocon protein, (for bacteria grown in F-media) also reflected that curcumin increases SPI2 expression upto 4–5 h (Figure 3A). As PhoPQ regulates SPI2 genes, it was speculated that curcumin acts via PhoPQ. When *phoP^-^* strain was used, there was no difference in the activity of SPI2 gene (*spiC*) on curcumin treatment, as assessed by β-galactosidase assay (Figure 3A). Also, the intracellular proliferation of curcumin treated and untreated *phoP^-^* strain was similar (Figure 3B) indicating that curcumin might be regulating SPI2 gene through PhoPQ. Thus, the increased proliferation of curcumin-treated bacteria in macrophages could at least be partially attributed to the up-regulation of SPI2 genes (at least *sseB* and *spiC*) by curcumin.

### Iron chelating property of curcumin partially protects *S.* Typhimurium against ROS and RNI by regulating *mntH* and *sitA*

**Figure 4:**
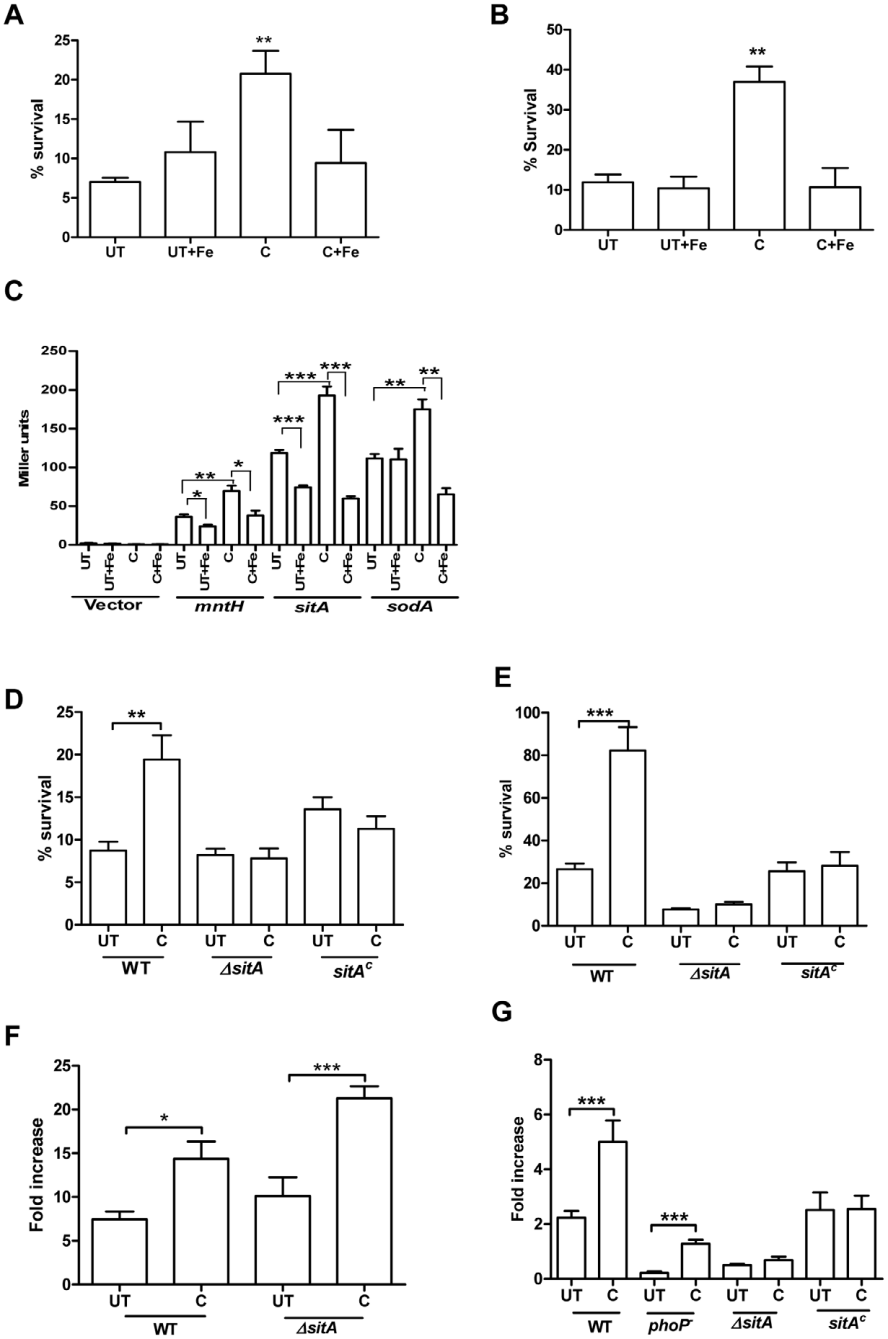
Regulation of antioxidant genes by curcumin to improve resistance of bacteria against oxidative stress. Survival of *S.* Typhimurium grown in presence or absence of curcumin (20 µM) and iron (32 µg/ml FeCl_3_ or 20 µM FeSO_4_) against1 mM H_2_O_2_ (**A, D**) or NaNO_2_ (**B, E**). **C.** Effect of curcumin on the transcriptional activities of *mntH, sitA, sodA* genes. β-galactosidase assay was performed 6 h post-incubation of *S.* Typhimurium harboring either *sitA* or *sodA-lacZ* construct in presence or absence of curcumin (20 µM) and iron (32 µg/ml FeCl_3_ or 20 µM FeSO_4_) in LB. **F & G.** Intracellular proliferation of curcumin treated and untreated bacteria in RAW 264.7 and NRAMP^+/+^ BMDM respectively. *** P<0.001, ** 0.001≤P<0.01 and * 0.01≤P<0.05. UT- untreated, C- curcumin treated, *ΔsitA* – *sitA* knockout and *sitA^c^* – *sitA* complement in *ΔsitA*.

Phagocytes exhibit their antimicrobial activity through the production of ROS and RNI. The abilities of curcumin treated and untreated bacteria to protect themselves from the cell damaging intermediates, ROS (H_2_O_2_) and RNI (NO^.^) were compared. NaNO_2_ protonates into HNO_2_, which rapidly dismutates to produce several nitrite species including NO^.^ [33]. The toxicity of NaNO_2_ solution is maximum in pH 4.4–5.5 range [34]. At low pH, H_2_O_2_ is quite stable and toxic. Survival of the bacteria was evaluated in the presence of either 1 mM H_2_O_2_ or NaNO_2_ at pH 5. Curcumin treated *S*. Typhimurium showed 2–3 fold higher survival in the presence of H_2_O_2_ and NO^.^ as compared to untreated *S*. Typhimurium (Figure 4A & B respectively).

MntH and SitABCD, iron and manganese transport systems are required for the resistance against H_2_O_2_ and virulence of *S*. Typhimurium [35], [36], [37] especially in NRAMP^+/+^ mice [38]. Manganese is required for the catalysis of enzymes like superoxide dismutase (SodA) [37], involved in resistance to the early oxygen-dependent microbicidal mechanisms of phagocytes [39]. The regulation of *sitA*, *mntH* and *sodA* by curcumin was determined by promoter assay. Promoters of respective genes were cloned upstream of the promoterless lacZ gene in pHG86 plasmid and the β-galactosidase assay was carried out to determine the change in promoter activity on curcumin treatment. Curcumin increased the promoter activity of these genes (Figure 4C). In addition, curcumin treatment did not alter the resistance of the Δ*sitA* strain against H_2_O_2_ and NO^.^ (Figure 4D & E) implicating that curcumin increases resistance of the bacteria against oxidative stress by regulating the expression of *sitA* and possibly *mntH*. As compared to untreated *ΔsitA* the fold proliferation of curcumin treated *ΔsitA* was high in RAW 264.7 (Figure 4F) cells but was indifferent in NRAMP^+/+^ BMDM (Figure 4G). The complement of *ΔsitA* strain behaved similar to wildtype. However, curcumin treated and untreated complement bacteria behaved similarly (Figure 4G) as the *sitA* gene was cloned without the promoter sequence. Curcumin treated *phoP^-^* bacteria showed higher fold proliferation in NRAMP^+/+^ cells indicating that curcumin also acts on pathways other than PhoPQ (may be *sitA, mntH* and *sodA*) to modulate intracellular survival of the bacteria in NRAMP^+/+^ cells. This suggests that the enhanced proliferation of curcumin treated bacteria in RAW 264.7 cells is not through the regulation of *sitA*. However, the regulation of *sitA* by curcumin may play a role on intracellular proliferation of bacteria in NRAMP^+/+^ host cells.

The iron binding property of curcumin has been implicated to play a role in the treatment of cancer and neurodegenerative disorders [40], [41], [42]. When tested, we found that iron chelation by curcumin had a role in partially protecting *S*. Typhimurium against H_2_O_2_ and NO^.^ toxicity. Supplementation of iron (32 µg/ml of FeCl_3_ or 20 µM FeSO_4_) in the growth media caused reversal of the phenomena observed in the presence of curcumin (Figure 4 A–C) implicating that iron chelating property of curcumin might regulate the antioxidant genes (*sitA* and *mntH*) and improve resistance against oxidative stress.

### Increased expression of pmr genes by curcumin protects the bacteria against AMPs

**Figure 5:**
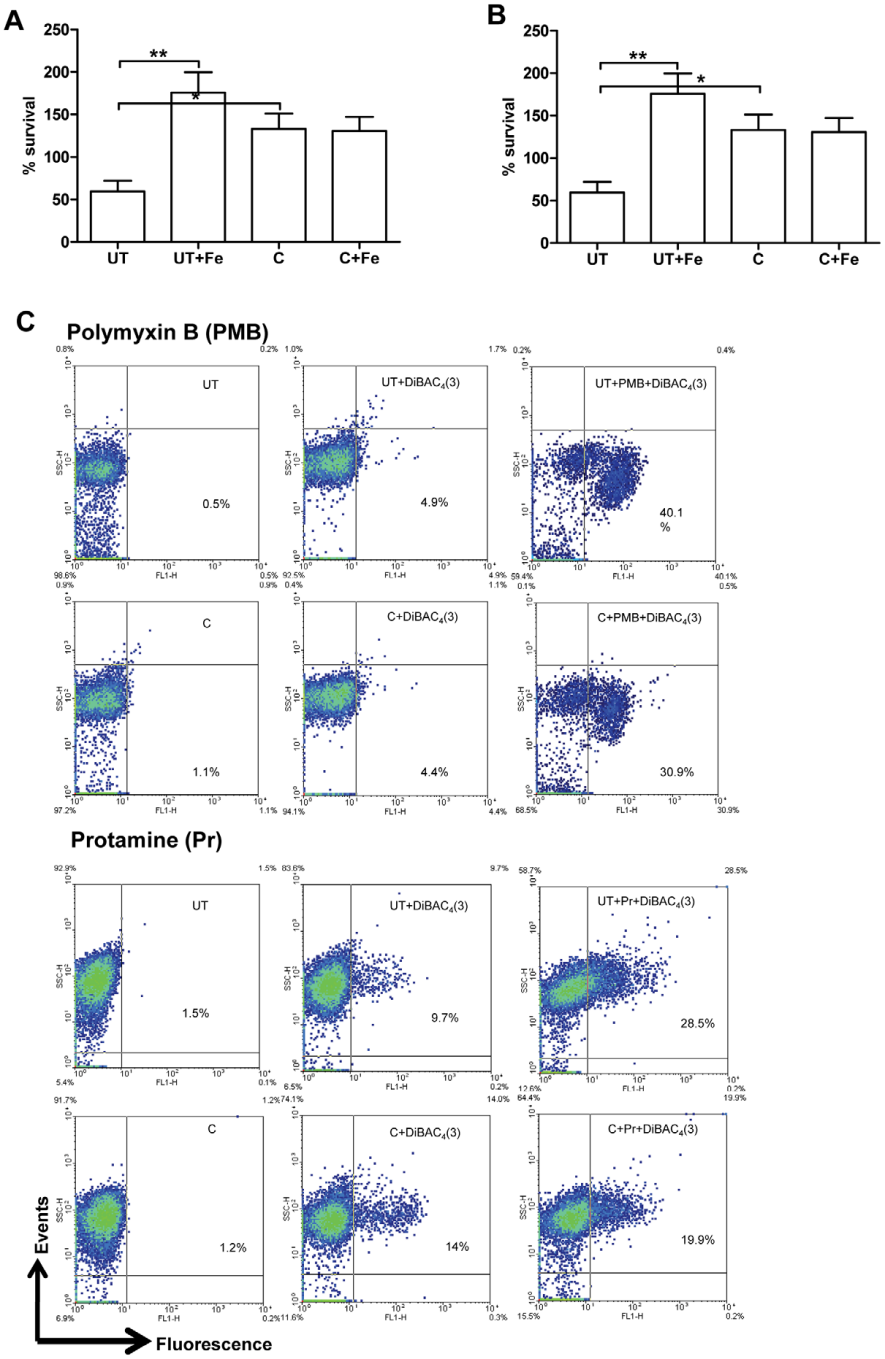
Sensitivity of *S*. Typhimurium to antimicrobial peptides. Survival of *S.* Typhimurium grown in presence (C) or absence (UT) of curcumin (20 µM) and iron (32 µg/ml FeCl_3_ or 20 µM FeSO_4_) against **A.** Polymyxin B (1 µg/ml) and **B.** Protamine (50 µg/ml). **C.** Membrane potential assay. The polymyxinB or protamine treated *S*. Typhimurium were stained with DiBAC_4_ (3) and analysed in FACS scanner.

Intestine 407 cells, derived from embryonic intestinal cells are rich producers of AMPs. To account for the improved survival of curcumin-treated *S*. Typhimurium in these cells (Figure 1), the sensitivity of the bacteria to AMPs (polymyxin B and protamine) was evaluated by CFU and flow cytometry. AMP attack leads to change in membrane permeability. This change in membrane permeability was quantified using a fluorescent, membrane potential sensitive dye, DiBAC_4_ (3). Curcumin-treated *S*. Typhimurium showed greater survival on AMP treatment (Figure 5 A & B) and exhibited greater membrane integrity as demonstrated by the decreased uptake of DiBAC_4_ (3) (Figure 5C).

**Figure 6:**
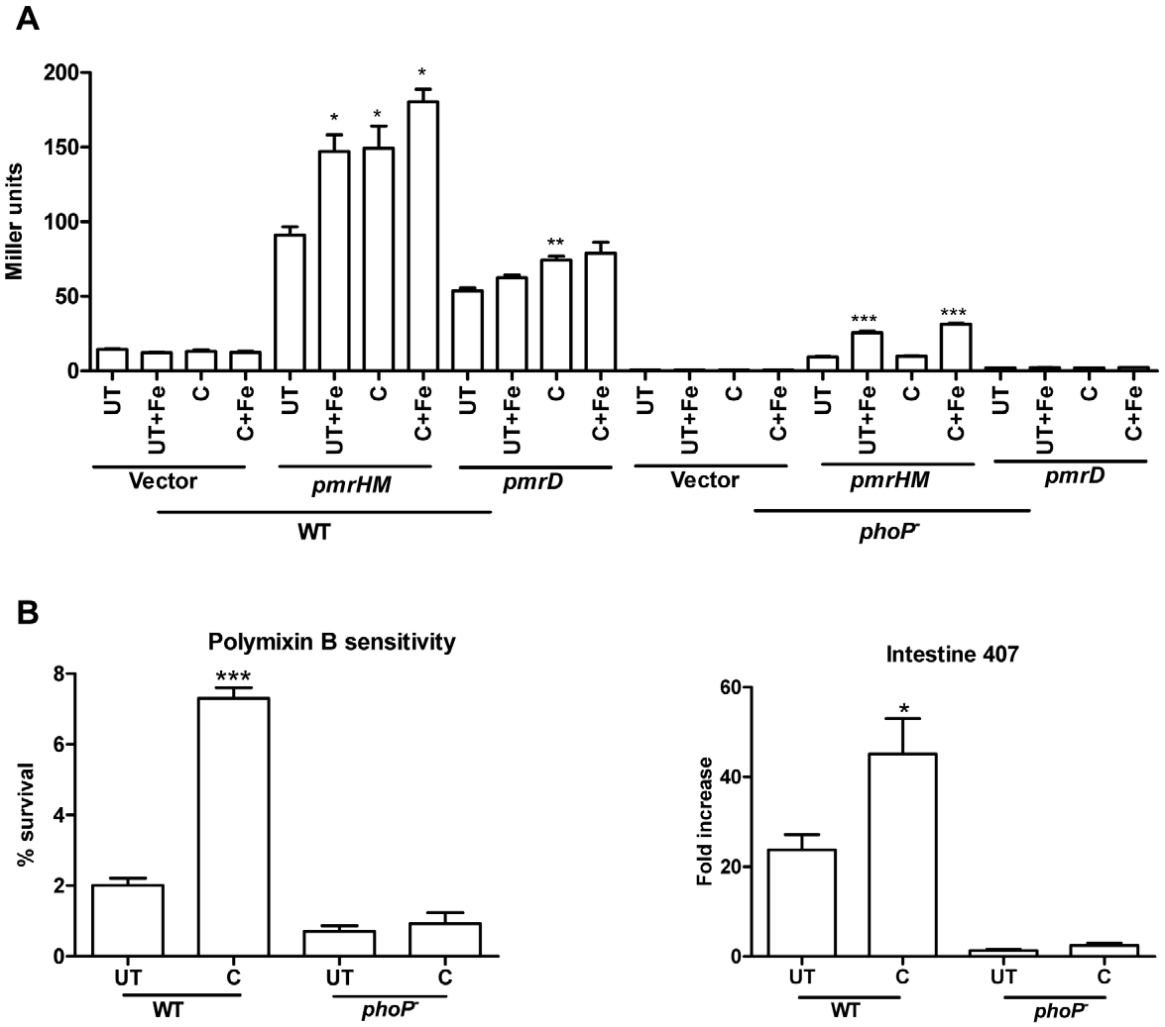
Regulation of *pmr* genes by curcumin to improve resistance of bacteria against AMPs. **A.** Effect of curcumin on the transcriptional activities of *pmrD* and *pmrHFIJKLM* genes. β-galactosidase assay was performed 3 h post-incubation of *S.* Typhimurium harboring either *pmrD* or *pmrHFIJKLM -lacZ* construct in presence or absence of curcumin (20 µM) and iron (32 µg/ml FeCl_3_ or 20 µM FeSO_4_) in LB. **B.** Susceptibilty of *phoP^-^* strain to polymyxin B. **C.** Intracellular survival assay of *phoP^-^* strain in Intestine 407 cells. The Intestine 407 cells infected with solvent (S), curcumin (C, 20 µM) treated or untreated (UT) *S.* Typhimurium were lysed at 2 h & 16 h post infection and fold replication of the bacteria was calculated. *** P<0.001, ** 0.001≤P<0.01 and * 0.01≤P<0.05.

*S.* Typhimurium protects itself from AMPs via different mechanisms, one of them being the modification of LPS residues reducing the net negative charge on the cell surface [43]. PmrAB and PhoPQ regulatory system sense specific environmental cues (low pH, low Mg^2+^, high Fe^3+^) and upregulate the genes involved in LPS modification like *pmrD, pmrHM, pmrE, pmrC, cld*. [15], [43]. High iron (Fe^3+^) activates PmrAB which in turn upregulates *pmrHM* and *pmrE*. The iron chelation caused by curcumin should lead to down-regulation of *pmrHM, pmrC* etc. The promoters of *pmrD* and *pmrHM* were cloned upstream of the promoterless lacZ gene in pHG86 plasmid. *S*. Typhimurium harboring *pmrD* and *pmrHM*-*lacZ* constructs were used to evaluate the change in promoter activity of these genes on curcumin treatment. We surprisingly found that curcumin increased the promoter activity of *pmrD* and *pmrHM* operon (Figure 6A). Hence, we hypothesized that curcumin might regulate *pmrD* and *pmrHM* through PhoPQ and not through PmrAB. The *pmrD* and *pmrHM*-*lacZ* fusions were transformed into *phoP^-^* strain and the promoter activity of respective genes was analysed on curcumin treatment. We found that curcumin failed to increase the promoter activity of *pmrD* and *pmrHM* in *phoP^-^* (Figure 6A) bacteria. It did not alter the susceptibility of *phoP^-^* to polymyxinB nor did it improve the survival of *phoP^-^* in Intestine 407 cells (Figure 6B) indicating that curcumin might regulate the expression of *pmr* genes through PhoPQ. The increased expression of *pmrD* and *pmrHM* may be the reason for the improved resistant (through LPS modification) of curcumin treated-bacteria against polymyxinB.

### Down-regulation of SPI1 genes by curcumin leads to reduced entry of *S.* Typhimurium into the epithelial cells

**Figure 7:**
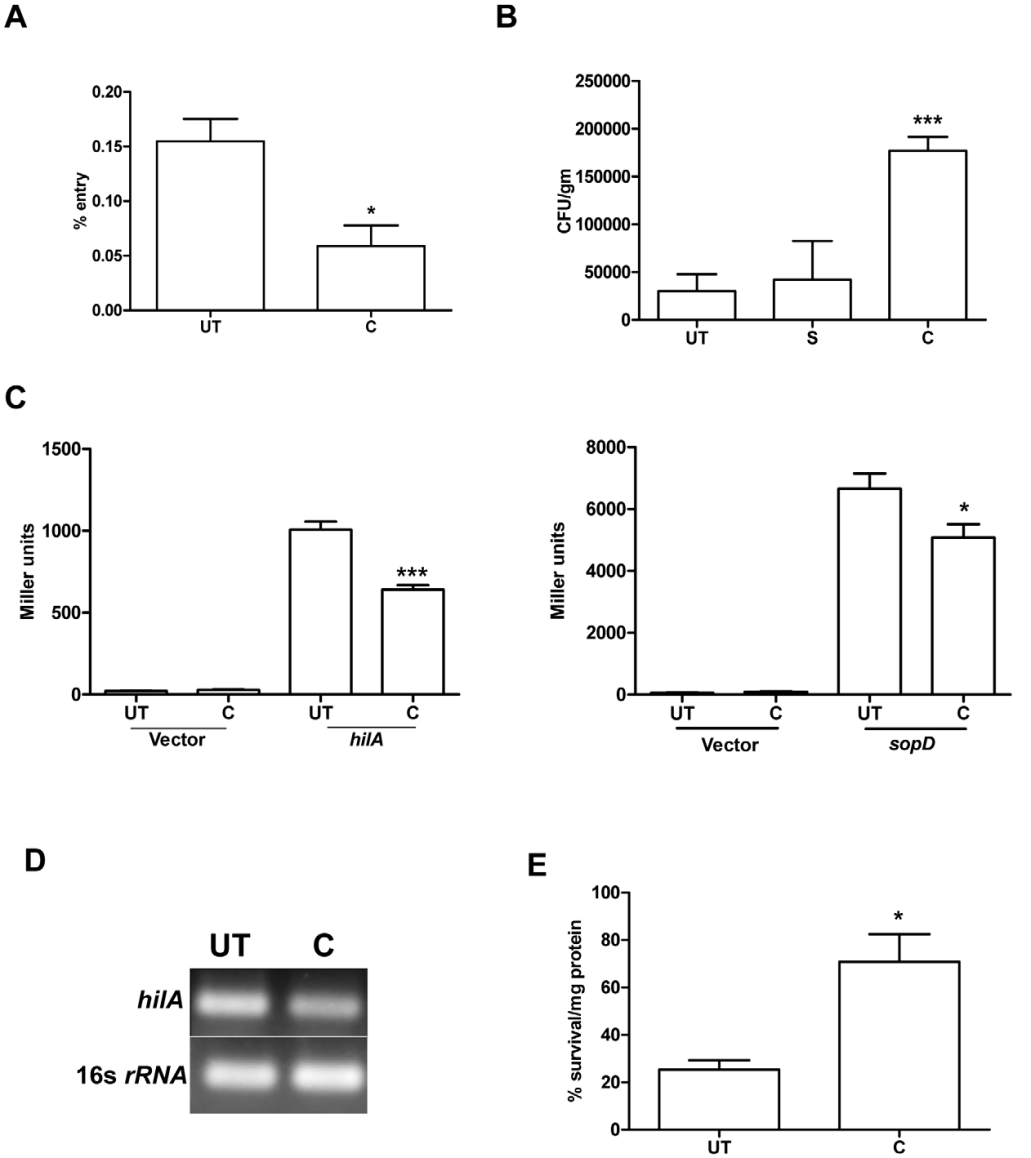
Curcumin treated bacteria show reduced entry in-vitro but not in-vivo. **A.** The entry of curcumin (C, 20 µM) treated and untreated (UT) *S*. Typhimurium in Intestine 407 cells. The entry in Intestine 407 cells was determined by lysing the infected cells 30 min post-infection. **B.** CFU per gram weight of Peyer's patch of mice infected intragastrically with curcumin-treated and untreated *S*. Typhimurium. 1 h post-infection Peyer's patch were aseptically isolated, processed to get CFU/gm. **C.** Effect of curcumin on the transcriptional activity of *hilA* and *sopD* promoter. β-galactosidase activity was performed using *S.* Typhimurium harboring either *hilA* or *sopD -lacZ* construct grown in presence or absence of curcumin to know the transcriptional activity. **D.** RT-PCR analysis to check the expression of *hilA* in curcumin-treated and untreated *S*. Typhimurium. RT-PCR with the mRNA isolated from curcumin treated and untreated *S*. Typhimurium was performed. **E.** Percentage survival of bacteria in the mucus from small intestine of mice. Curcumin-treated and untreated bacteria were inoculated in the mucus for 1 h and their survival calculated. *** P<0.001 and * 0.01≤P<0.05.

Even though curcumin treatment increased the pathogenicity of *S*. Typhimurium, the treated-bacteria were defective in entry into the host cells *in vitro* (Figure 7A). This defect in entry could be due to downregulation of SPI1 genes required for entry into non-phagocytic host cells. HilA is a master regulator of SPI1 genes like *sipB, sipC*, *sopD*, *sopB* etc. (24). *hilA* is also required by the bacteria to colonize in the extracellular luminal compartment of the intestine [44].*S*. Typhimurium harboring *hilA* and *sopD-lacZ* fusions were constructed as described previously [20]. The change in promoter activity of these genes on curcumin treatment was examined by β-galactosidase assay. Curcumin indeed decreased the promoter activity of the SPI1 genes tested.Thus the defect in invasion could be ascribed to the down-regulation of SPI1 genes (*hilA* and *sopD*) by curcumin (Figure 7C & D). Down-regulation of *hilA* should result in reduced bacterial load in the small intestinal epithelia. Nevertheless, to our surprise we found that, 1 h post-intragastric infection, the bacterial load was high in the PP (small intestine) of the mice infected with curcumin-treated *S*. Typhimurium (Figure 7B).

Bacteria face a myriad of environmental stress during their passage through the gastrointestinal tract to reach the intestine, where they invade the M cells and epithelia of the small intestine. In the intestine within the mucus, they encounter a plethora of antimicrobial agents. Hence, the survival efficacy of curcumin treated-bacteria was tested in the mucus isolated from the small intestine of mice. The treated-bacteria showed higher survival in mucus (Figure 7 E). This improved survival against antimicrobial agents could lead to increased number of bacteria reaching the intestinal epithelia and invading it.

Thus, curcumin enhances the pathogenicity of *S*. Typhimurium by increasing its resistance against different immune components.

## Discussion

The intracellular survival assay demonstrated that curcumin treatment improved the pathogenicity of *S*. Typhimurium. The ability of *S*. Typhimurium to survive the oxidative stress inside the SCV determines the progression of disease [23], [29]. SPI2 genes play a very important role in determining this progression [45]. It interferes with the cellular functions and the innate immune components of the host. The increased activity of SPI2 genes in curcumin treated bacteria during the initial 4–5 h might accelerate the progression of disease. The improved pathogenicity of curcumin-treated *S*. Typhimurium can also be ascribed to its increased resistance against the damaging effects of the oxidative congeners, H_2_O_2_ and NO^.^, and AMPs (polymyxin B and protamine).

*S*. Typhimurium harbors quite a few mechanisms to counteract oxidative stress. One such mechanism is through the acquisition of Mn^2+^ ions. Horsburgh *et al.* demonstrated that accumulation of manganese in the bacterial cell catalytically detoxifies reactive oxygen species and protects the bacteria from the oxidative damage [46]. It is known that mutants of *sitABCD* and *mntH* are attenuated for the survival within the host [35], [37], [47] and are required for full virulence especially in NRAMP1^+/+^ mice [38]. *S*. Typhimurium also has SodA which is important for resistance against early oxygen dependent killing in macrophages and protection against oxidative stress under iron limiting conditions [39]. Our work demonstrate that curcumin increase the expression of *sitA, mntH* and *sodA* conferring protection against oxidative stress. Curcumin also downregulates *hilA* which correlates well with the findings of Ellermeier *et al.* where they show that addition of metal chelator 2,2-dipyridyl to the growth medium increase the expression of *sitA* and decrease the expression of *hilA* [48]. It is also known that *sitA* and *mntH* are regulated by iron concentration [14] and cation chelators [36], [49]. As curcumin acts as an iron chelator [40], [41] we implicate that the iron chelation caused by curcumin may modulate the expression of the genes, *sitA* and *mntH,* protecting the bacteria from oxidative stress, and *hilA,* hindering its entry into epithelial cells. Iron chelators are known to govern the expression of *phoP* and other SPI2 genes [47]. The regulation of SPI2 genes (atleast *spiC* and *sseB*) by curcumin could also be explained through the iron chelation property of curcumin. Zaharik *et al.* showed that depletion of Fe^2+^ and Mn^2+^ in SCV causes up-regulation of *mntH* and *sitA*. Martin-Orozco *et al.* demonstrated that NRAMP1 has no effect on PhoP induction and the bacteriostatic/cidal effect of NRAMP1 is independent of PhoPQ. Our result show that curcumin has no effect on intracellular proliferation of *ΔphoP* in RAW 264.7 cells but increases its proliferation in NRAMP1^+/+^ cells, as compared to untreated bacteria. Curcumin does not alter the proliferation of *ΔsitA* in both RAW 264.7 cells and NRAMP^+/+^ BMDM. This suggests that the action of curcumin on *sitA* is also important for the survival of bacteria especially in NRAMP1^+/+^ cells.

The ability of curcumin to scavenge free radicals might also offer protection against oxidative stress [50]. Curcumin was found to be incorporated into *S.*Typhimurium (Materials and Methods S1, 50 to 75 µg/gm of bacterial dry mass, Supplementary Figure S3, Previous report suggest that iron chelators that penetrate bacteria (dipyridyl, o-phenanthroline, and desferrioxamine) can protect the DNA from damage by exogenous H_2_O_2_ [51]. Similarly, curcumin that enters the cell might also protect the bacteria against the oxidative stress.

*Salmonella* counteracts the effect of AMPs either by LPS modification or secretion of some proteases [43]. PhoPQ and PmrAB regulate the LPS modifying system. These two-component regulatory systems are activated by different environmental cues. Upon activation, PhoPQ increases the transcription of *pmrD* whose protein product stabilizes activated PmrA which in turn activates the genes involved in LPS modification, e.g. *pmrHM, pmrE* etc (22). PmrA is also activated independently by high Fe^3+^/Al^3+^, low pH etc. [15]. Iron chelation caused by curcumin should lead to down-regulation of PmrAB system and hence *pmrHM, pmrE* etc. However, we found that in presence of curcumin *pmrD* and *pmrHM* are upregulated.

Curcumin has been shown to modify the function of membrane proteins by changing the lipid bilayer properties [52]. In a similar way, curcumin might modulate the function of PhoQ, a membrane bound sensor kinase that is activated upon conformational change [53], further leading to the enhanced transcription of *pmrD* and hence the activation of LPS modifying system. Iron chelators increase the expression of *phoP* [47]. Iron chelation by curcumin might also contribute to the above phenomena. Further study needs to be done to elucidate the exact mechanism by which curcumin might regulate PhoPQ system. It is also known that activation of PhoPQ leads to the down-regulation of *hilA* and other SPI1 genes, [54], [55], [56] which conforms to our data of down-regulation of *hilA* by curcumin.

**Figure 8:**
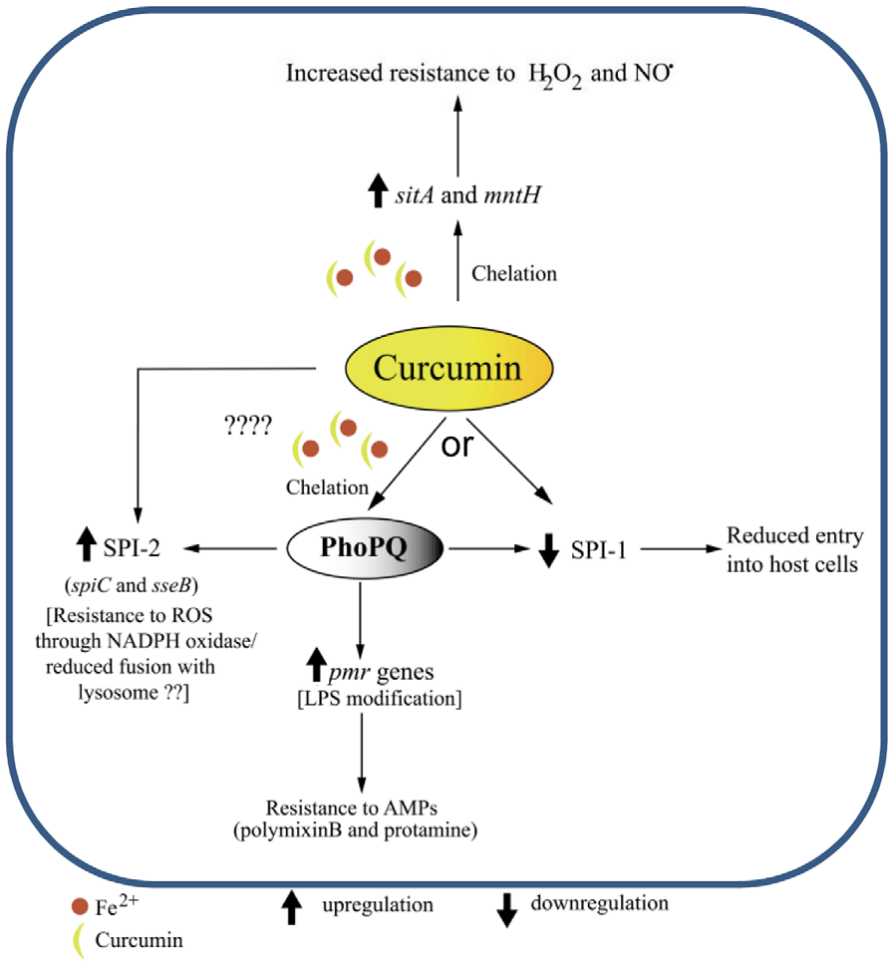
Schematic summary representing the mode of action of curcumin. Iron chelation caused by curcumin might increase the expression of different genes involved in defense of *S*. Typhimurium against host immune response.

Curcumin-treated WT bacteria but not *ΔphoP* (data not shown) are defective in entry into the epithelial cells under *in vitro* condition. However, 1 h post-intragastric infection, higher numbers of bacteria were found in PP of mice infected with curcumin treated bacteria. The resistance offered by curcumin against different antimicrobial agents might benefit the bacteria when inside intestinal lumen where it encounters hoard of antimicrobials. This may result in increased number of bacteria crossing the mucus barrier and invading the M-cells in PP. Further, the bacterial load was high in the different organs of the mice infected (intragastrically or intraperitoneally) with curcumin treated *S*. Typhimurium. This corroborates with the observations that curcumin protects *S*. Typhimurium against the innate immune components of the host viz. ROS, RNI and AMPs that the bacteria encounter in the intestinal lumen, macrophages and neutrophils that disseminate them to different systemic organs. The schematic summary for the possible mode of action of curcumin is given in Figure 8.

Curcumin treated *Salmonella enterica* serovar Typhi also shows higher fold proliferation in Intestine 407 and RAW 264.7 cell-lines (Supplementary Figure S4). Treatment of RAW 264.7 cells with curcumin (20 µM) also lead to increased intracellular proliferation of *S*. Typhimurium (S. A. Marathe and D. Chakravortty, unpublished data) indicating that treatment of either pathogen or host with curcumin enhances its pathogenicity.

Turmeric is widely used as a therapeutic as well as a food ingredient especially in Asia [1]. Certain Southeast Asian communities consume 1.5 g/person/day of turmeric that corresponds to 0.03–0.12 g of curcumin/person/day [1]. According to Centers for Disease Control and Prevention, the prevalence of *Salmonella* infection is more in Asia, Africa and Latin America. In addition, the prevalence of typhoid is highest in Asia. Of 2,16,000 deaths due to typhoid in the year 2000, more than 90% of morbidity and mortality occurred in Asia [57].We hypothesize that the high consumption of curcumin could be one of the reason for the widespread occurrence of *Salmonella* infections in Asian countries (especially in Southeast Asia). Even though curcumin has protective action against cancer [1], [42] and *H.pylori* infections [58] it should be consumed with a caution especially during the outbreak of *Salmonella* infections and in the endemic areas. Curcumin is not a panacea for all! Our data is the first of its kind which suggest that the curcumin can increase the pathogenicity of *Salmonella* by making it more robust. Hence, during *Salmonella* infection, the consumption of curcumin should be avoided.

## Supporting Information

Figure S1 Cytotoxicity of curcumin. A. MTT test for cytotoxicity in RAW 264.7, Intestine 407 and Caco-2 cells after 24 h of curcumin (20 µM) treatment. B. Growth curve in LB media: The growth pattern of *S.* Typhimurium was checked in presence or absence of curcumin (20 M).
(0.07 MB TIF)

Figure S2 Growth curve of *S.* Typhimurium in F-media. *S.* Typhimurium grown overnight in LB, either in presence (C, 20 µM) or absence of curcumin (UT) was subcultured in F-media, pH 5, incubated at 37°C under shaking conditions and the OD measured at 600 nm at different time points and plotted.
(0.03 MB TIF)

Figure S3 Incorporation of curcumin in *S.* Typhimurium. *S.* Typhimurium grown in presence or absence of curcumin (C, 20 µM) was pelleted and then washed twice with PBS. The pellet was dried and weighed. The dried pellet was resuspended in DMSO to dissolve curcumin present, if any. The Absorbance of the solution was taken at 420 nm. The weight of curcumin per gram weight of bacterial dry pellet was analysed.
(0.04 MB TIF)

Figure S4 Fold proliferation of *S.* Typhi. The cells (RAW 264.7 and Intestine 407) infected with curcumin (C, 20 µM) treated and untreated (UT) *S.* Typhimurium were lysed at 2 h & 16 h post infection. The fold replication of the bacteria from 2 h to 16 h was calculated.
(0.05 MB TIF)

Table S1 Primers used in this study.
(0.04 MB DOC)

Materials and Methods S1 Supplementary materials and methods.
(0.02 MB DOC)
