# Supplementary figures and images for: Curcumin Increases the Pathogenicity of Salmonella enterica Serovar Typhimurium in Murine Model
Source: PLoS One. 2010 Jul 9;5(7):e11511. doi: 10.1371/journal.pone.0011511 (PMC2901387; doi:10.1371/journal.pone.0011511)

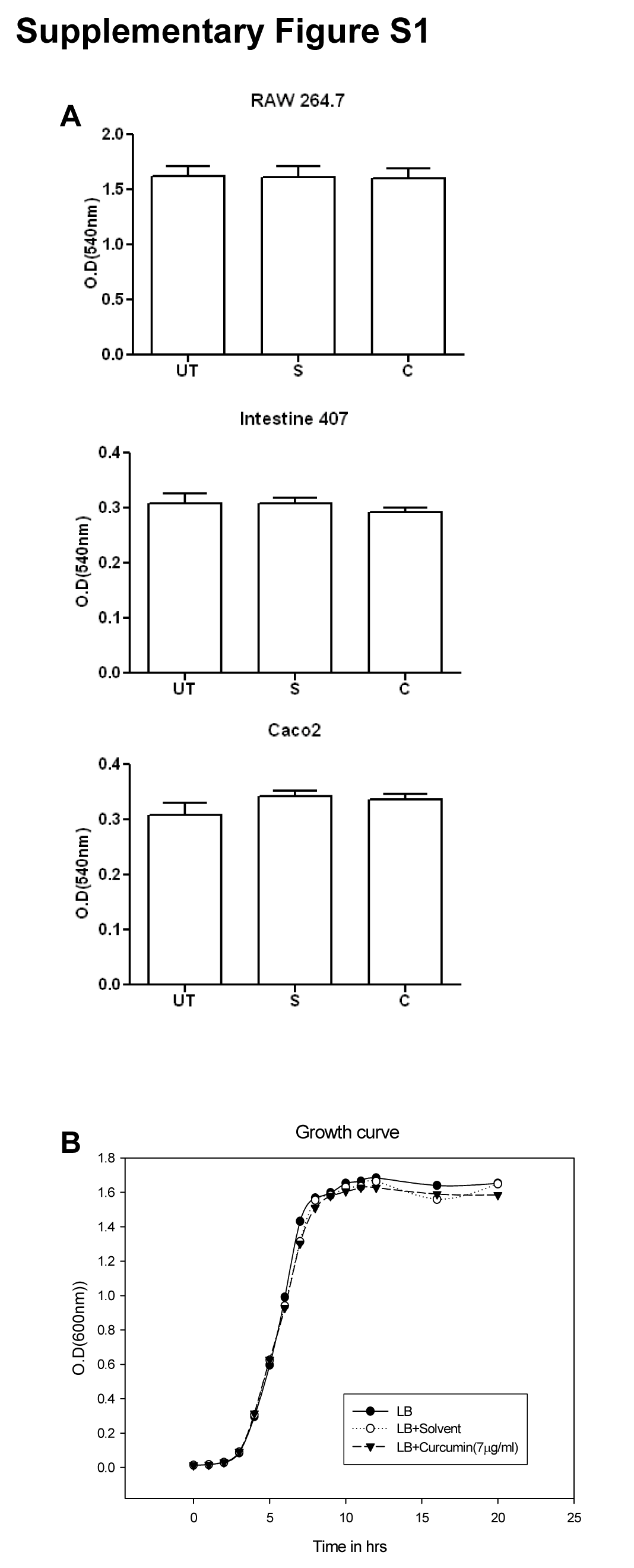

Supplement: Figure S1 — Cytotoxicity of curcumin. A. MTT test for cytotoxicity in RAW 264.7, Intestine 407 and Caco-2 cells after 24 h of curcumin (20 µM) treatment. B. Growth curve in LB media: The growth pattern of S. Typhimurium was checked in presence or absence of curcumin (20 M). (0.07 MB TIF) [file pone.0011511.s001.tif]

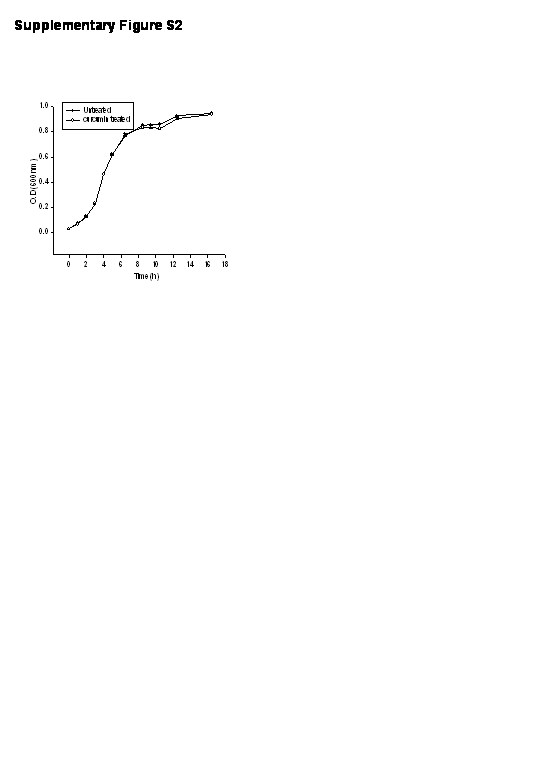

Supplement: Figure S2 — Growth curve of S. Typhimurium in F-media. S. Typhimurium grown overnight in LB, either in presence (C, 20 µM) or absence of curcumin (UT) was subcultured in F-media, pH 5, incubated at 37°C under shaking conditions and the OD measured at 600 nm at different time points and plotted. (0.03 MB TIF) [file pone.0011511.s002.tif]

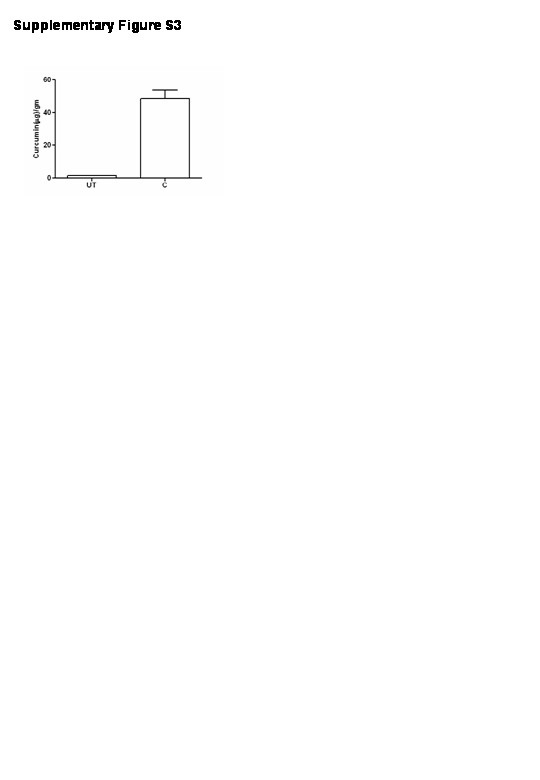

Supplement: Figure S3 — Incorporation of curcumin in S. Typhimurium. S. Typhimurium grown in presence or absence of curcumin (C, 20 µM) was pelleted and then washed twice with PBS. The pellet was dried and weighed. The dried pellet was resuspended in DMSO to dissolve curcumin present, if any. The Absorbance of the solution was taken at 420 nm. The weight of curcumin per gram weight of bacterial dry pellet was analysed. (0.04 MB TIF) [file pone.0011511.s003.tif]

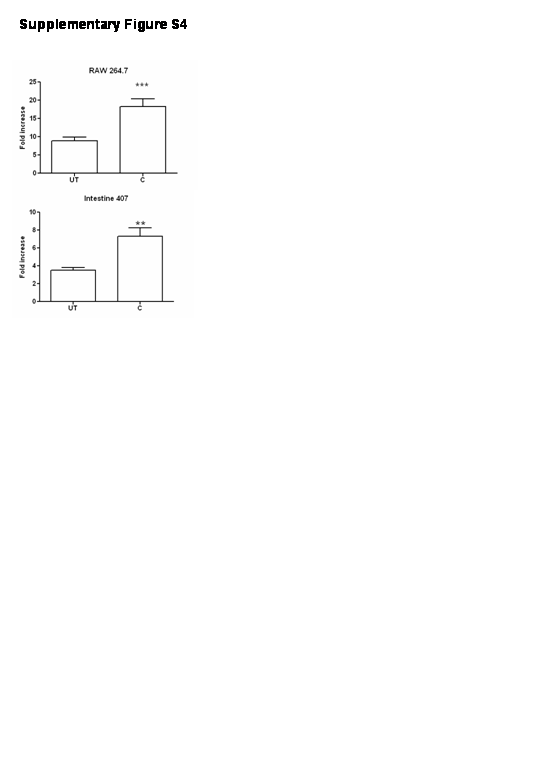

Supplement: Figure S4 — Fold proliferation of S. Typhi. The cells (RAW 264.7 and Intestine 407) infected with curcumin (C, 20 µM) treated and untreated (UT) S. Typhimurium were lysed at 2 h & 16 h post infection. The fold replication of the bacteria from 2 h to 16 h was calculated. (0.05 MB TIF) [file pone.0011511.s004.tif]
