# Supplementary material for: Curcumin Increases the Pathogenicity of Salmonella enterica Serovar Typhimurium in Murine Model
Source: PLoS One. 2010 Jul 9;5(7):e11511. doi: 10.1371/journal.pone.0011511 (PMC2901387; doi:10.1371/journal.pone.0011511)
Supplement: Table S1 — Primers used in this study. (0.04 MB DOC) [file pone.0011511.s005.doc]

Supplementary Table S1 – Primers used in this study

| Primers | Sequence 5′ - 3′ |
| --- | --- |
| *sitA K/O* forward | atgacgaatctacatcgtctgaaaacactcctgattgccggtgtaggctggagctgcttc |
| *sitA K/O* reverse | tcattgttgactcctcagtccgtcgttaatgccgttgacgcatatgaatatcctcctta |
| *sitA cloning* forward | agtccggatccatgacgaatctacatcgtct |
| *sitA cloning* reverse | actgaagcttattgactcattgttgactcct |
| *hilA* forward | ttaacatgtcgccaaacagc |
| *hilA* reverse | gcaaactcccgacgatgtat |
| *16S rRNA* forward | gatcatggctcagattgaacgctggcgg |
| *16S rRNA* reverse | caccgctacacctggaattatacccccctc |
| *mntH* forward* | agtcgaattccgtgcacattctatgcaacagc |
| *mntH* reverse* | agtcggatccgcctcaaaaacatagcctttgc |
| *hilA* forward* | agtcgaattccccggcgctgactctctctgca |
| *hilA* reverse* | agtcggatccagtgtattctcttacagggtg |
| *sopD* forward* | agtcgaattcggaagaagcgctcaaagaggc |
| *sopD* reverse* | agtcggatcctccttcaaatttatattattc |
| *sitA* forward* | agtcgaattcgtgggcactggctttcataca |
| *sitA* reverse* | agtcggatccagtatccctcgcaacaatgtgg |
| *sodA* forward* | agtcgaattccgggaatgtaattgcagtgtg |
| *sodA* reverse* | agtcggatcccatctccattattgtcgagcg |
|  |  |

***** represents primers for cloning promoter region
