# Supplementary material for: Curcumin Increases the Pathogenicity of Salmonella enterica Serovar Typhimurium in Murine Model
Source: PLoS One. 2010 Jul 9;5(7):e11511. doi: 10.1371/journal.pone.0011511 (PMC2901387; doi:10.1371/journal.pone.0011511)
Supplement: Materials and Methods S1 — Supplementary materials and methods. (0.02 MB DOC) [file pone.0011511.s006.doc]

**Supplementary Material and Methods S1**

*Cytotoxicity assay by MTT***.** (3-(4, 5-dimethylthiazole-2-yl)-2, 5-diphenyl tetrazolium bromide)- 1–3 X10^4^ cells per well were seeded in 96-well plates and incubated overnight at 37°C, 5 % CO_2_ to allow the cells to adhere. Medium was replaced with fresh medium containing different concentration (2, 5 and 7 µg/ml) of curcumin. Medium from the wells was removed after 24 h and 100 µl MTT (250 μg/ml) containing medium was added per well. The plate was incubated for 1-4 h at 37°C and 5% CO_2_. MTT containing medium was removed followed by addition of 110 µl dimethyl sulfoxide (DMSO) to each well. The crystals were dissolved and 100 µl of DMSO was taken from each well in another 96-well plate. The plate was read at 540 nm in ELISA microplate reader (ELx 800 MS).

*Growth curve in LB media-**S.* Typhimurium were grown in 25 ml LB in presence or absence of curcumin (7 μg/ml) at 37°C in duplicates and optical density (OD) at 600 nm was taken at indicated time points and plotted on the graph.

*Curcumin incorporation in bacteria-* *S*. Typhimurium in late log-phase were pelleted down. The pellet was washed twice with PBS, dried, weighed and then disrupted. The disrupted pellet was resuspended in DMSO to dissolve curcumin. The solution was centifuged at 10,000 rpm to settle down the debris. Absorbance of the supernatant was taken at 420 nm. The amount of curcumin in each sample was obtained using a standard curve for curcumin (OD verses weight).
